# Ex vivo engineering of neural tissue structure and growth using sequential 2D and 3D solid scaffolds

**DOI:** 10.1007/s11626-026-01182-y

**Published:** 2026-05-12

**Authors:** Orly E. Weiss, Danny Baranes

**Affiliations:** 1 Department of Molecular Biology, Ariel University, Ramat HaGolan 65, Ariel, Israel; 2 The Dr. Miriam and Sheldon G. Adelson School of Medicine, Department of Medical Sciences, Ariel University, Ariel, Ramat HaGolan 65, Israel

**Keywords:** Neural tissue, Coral skeleton, Aragonite, Hippocampal tissue culture, 3D neural tissue morphology

## Abstract

Brain injury disrupts tissue integrity, creating wounds with complex boundaries that hinder effective repair. Regeneration and reconnection require guiding deformed tissue along proper growth pathways. Pre-engineered scaffold implants made from biomaterials offer promise; however, while hydrogel-based scaffolds are common for brain repair, their low mechanical strength and slow cell growth limit effectiveness. This study examined solid scaffolds, which provide superior mechanical support and promote rapid cell growth, to modulate the growth behavior of injured hippocampal tissue. Three scaffold types were used: planar bioactive glass, planar aragonite (promoting neuronal and astrocytic growth), and three-dimensional glass beads. The scaffolds were applied in two steps. First, hippocampal tissue chunks from postnatal rat brains were cultured on the planar substrates; then, glass beads were added. On glass, tissue adopted a round/oval shape with pronounced vertical growth, while on aragonite it flattened and spread irregularly, reaching lengths twice as large and an area 3.8 times greater than on glass. In the second step, adding glass beads led to vertical growth on glass, with tissue encapsulating beads to form a complex 3D structure. In contrast, aragonite-supported tissue formed bump-like structures when encapsulating the beads, remaining largely planar. Also, they showed a tenfold lower bead density and twofold greater inter-bead distances than tissue on glass. In both cases, cellular outgrowth occurred. These findings show that sequential application of solid scaffolds with distinct structural properties can guide diverse tissue growth behaviors and serve as a strategy for fabricating neural implants, with implications for treating brain trauma and disease.

## Introduction

Implants for braintreatment after trauma or disease require a uniquely complex engineering approach due to the soft nature of brain parenchyma and its limited regenerative capacity. Consequently, scaffolds for brain implantation must both support and protect damaged tissue from further injury and accelerate tissue regeneration (Ahearne 2014). For example, these scaffolds should disrupt inhibitory astrocytic scar formation to allow axonal penetration while restraining harmful overactivation of astrocytes (Aurand *et al*. 2012; Anderson 2016), and while evoking minimal foreign body response (Barreto 2023). Immediate neuronal protection is essential to prevent the spread of secondary damage following the initial insult, and scaffolds must promote, in one approach through drug delivery (Carnicer-Lombarte *et al*. 2021), rapid yet controlled regeneration to restore neural function (Chróścicka 2016). Understanding the principles of engineering these specialized implants—and how brain tissue integrates with them—is critical. This dual focus not only informs the design of more effective brain scaffolds but also advances our overall approach to treating neural injuries, potentially improving outcomes in patients with traumatic brain injuries or neurodegenerative diseases.

Current tissue engineering approaches for brain wound repair have primarily employed soft hydrogel scaffolds (Fu *et al*. 2011; Frega *et al*. 2014; Cook 2017; Ghuman 2018). Hydrogels are favored for promoting cell proliferation and differentiation by entrapping cells—such as stem cells—and enabling the sustained, localized release of growth factors and other bioactive molecules (Hendler 2023; Hammam *et al*. 2024). In rodent models of stroke and traumatic brain injury, hydrogel-based implants have created a favorable microenvironment that stimulates neural regeneration and enhances repair (Karageorgiou and Kaplan 2005; Fu *et al*. 2011; Johnson *et al*. 2013; Cook 2017;  Jeyachandran and Cerruti 2023). However, the inherent softness of hydrogels poses a significant limitation: their low mechanical strength often results in structural instability. This instability, linked to scaffold-cell association (Kargozar *et al*. 2020), can lead to collapse under physiological stresses, potentially damaging surrounding tissue and hindering healing. Consequently, more robust implants—such as composite scaffolds or solid biomaterials—may be necessary to provide both structural support and promote neural growth, representing a promising direction for future brain tissue engineering research.

Solid matrices and scaffolds, like ceramics and rigid synthetic plastics or solid biomaterials, can support tissue growth, regeneration, and wound repair (Koyama and Ikegaya 2018; Lam *et al*. 2021; Kim *et al*. 2024), and have been used mainly for bone repair (Lee *et al*. 2014). Their superior mechanical strength provides robust support that enhances cell adhesion and growth (Kim *et al*. 2024). Unlike hydrogels, these materials offer long-term structural stability and can be engineered with specific surface features—consistent area, optimal roughness, and tailored charge—to further promote tissue growth. Additionally, solid scaffolds can be designed with custom three-dimensional structures that mimic the native configuration of wound void volume and injured tissues (Lizarraga-Valderrama 2020; Lam *et al*. 2021; Kim *et al*. 2024), allowing for precise tuning of cues necessary for optimal regeneration and damage repair.

Building on these robust examples of solid scaffolds, one promising material is bioactive glass, which has been traditionally used for bone repair (Lu *et al*. 2016; Kim *et al*. 2024) and is now emerging as a strong candidate for peripheral neural tissue engineering (Millet *et al*. 2010; Martino *et al*. 2018). Typically composed of borosilicate glass, bioactive glass is chemically inert and features surface silanol (Si–OH) groups that influence wettability and can be modified to enhance cell adhesion (Morad 2019, 2020). Manufactured as micro/nanoparticles and beads, this material reinforces soft scaffolds, thereby improving their structural stability (Negishi 2021). In vitro, flat bioactive glass coverslips support neuronal adhesion and promote growth in a planar configuration (Niu 2022; Nih *et al*. 2018), while glass beads facilitate the development of three-dimensional neuronal cultures (Peretz *et al*. 2007; Pautot *et al*. 2008), offering a more physiologically relevant environment.

Additionally, calcium carbonate, a solid scaffold material used in bone (Qu *et al*. 2019; Politrón-Zepeda *et al*. 2024) and cartilage repair (Qu *et al*. 2019), can enhance cell adhesion, proliferation, and differentiation into specific cell types (Sachlos and Czernuszka 2003; Shafiu Kamba and Zakaria 2014; Savya 2022). Notably, calcium carbonate exhibits significant regenerative potential in neural systems.

Aragonite, a crystalline form of calcium carbonate derived from marine coral skeletons, has been studied for its biocompatibility in biomedical applications, including bone tissue engineering and drug delivery (Shimba 2019). It supports both planar and semi-3D growth of dissociated hippocampal neurons, astrocytes, and tissue slices. For example, Peretz *et al*. demonstrated that 3D aragonite biomatrices significantly enhance neuron and astrocyte survival (Shimojo 2020). More recent studies by Hendler *et al*., Morad *et al*., and Weiss *et al*. further highlight the role of aragonite-polylysine scaffolds in modulating astrocytic reactivity and promoting regeneration (Sugimoto *et al*. 1998; Sutula 2002; Tanikawa 2023).

In conclusion, the structural integrity and bioactivity of both glass and aragonite emphasize the critical role of solid scaffolds in neural regeneration, ensuring mechanical stability, enhanced cell adhesion, and optimal conditions for restoring complex neural networks.

Motivated by the role that solid scaffolds play in neural regeneration, as outlined above, we investigated the structural and cellular behavior of hippocampal tissue explants on planar and 3D solid substrates—glass and aragonite. The goal was to gain insights that could enable precise control over tissue responses to engineered brain implants. Tissue engineering was performed in two distinct steps: first, the tissue was cultivated on a planar surface; then, glass beads (70-μm diameter) were added, resulting in 3D tissue growth on the beads. Tissue behavior on the planar surfaces differed, with compact vertical growth on glass and flattening on aragonite. Addition of glass beads further diversified tissue structure: on glass, the tissue encapsulated beads and expanded into complex 3D formations, whereas on aragonite it maintained a planar profile with localized bumps. Both substrates supported substantial cellular outgrowth from the tissue, highlighting their potential for regeneration. These findings highlight the potential to design tissue growth and morphological outcomes when exposed to preengineered solid implants. This approach holds significant clinical promise for enhancing neural tissue regeneration following brain injury or disease.

## Materials and methods

### Aragonite crystals cleaning procedure

Commercially sourced, naturally occurring aragonite particles (1.5–5.0 mm) were subjected to a cleaning process to remove organic residues. The particles were first treated with a 10% sodium hypochlorite solution (Sigma-Aldrich, St. Louise, MO) for 10 min, followed by a single rinse with double-distilled water (DDW). Next, they were immersed in a 1-M NaOH solution for 5 min and rinsed once with DDW. The samples were then placed in a 35% hydrogen peroxide (H₂O₂) solution (Romical, Toronto, Canada) for 10 min and washed three times with DDW. After cleaning, the samples were air-dried overnight at room temperature in a laminar flow hood and then autoclaved.

### Hippocampal tissue culture

Sterile 12-mm-diameter coverslips (used as controls) and aragonite pieces were coated with a 20 ng/ml poly-D-lysine solution (Sigma-Aldrich) 1 d before cell culture and incubated overnight at 4°C. The following day, the samples were washed twice with double-distilled water (DDW) and dried in a biological hood. Hippocampi from postnatal 1–3-d-old Sprague-Dawley rats (Envigo, Indianapolis, IN) were dissected and manually sectioned into fragments averaging 0.125 mm^3^ in size. A single hippocampal section was placed onto each coverslip or aragonite piece and incubated overnight in “First-day medium,” which consisted of 85.1% minimum essential Eagle’s medium (Sigma-Aldrich), 11.5% heat-inactivated fetal bovine serum (Biological Industries, Kibbutz Beit Haemek, Israel), 1.2% L-glutamine (Sigma-Aldrich), and 2.2% D-glucose (Sigma-Aldrich), maintained at 37 °C with 10% CO₂. The following day, the medium was replaced with a growth medium containing 45% minimum essential Eagle’s medium (Sigma-Aldrich), 40% Dulbecco’s modified Eagle’s medium (Sigma-Aldrich), 10% nutrient mixture F-12 Ham (Sigma-Aldrich), 0.25% (w/v) bovine serum albumin (Sigma-Aldrich), 0.75% D-glucose (Sigma-Aldrich), 0.25% L-glutamine (Sigma-Aldrich), 0.5% B-27 supplement (Gibco, Waltham, MA), 0.1% kynurenic acid (Sigma-Aldrich), 0.01% of 70% uridine (Sigma-Aldrich), and 30% 5-fluoro-2’-deoxyuridine (Sigma-Aldrich). On day 7 of cultivation, glass beads (Biospec Products; 0.1-mm diameter) were added to the wells at an average density of 0.005 beads/µm, and the tissue samples were further cultivated for a total of 22 d.

To ensure comparable initial adhesion, the same poly-D-lysine protocol was applied to both planar substrates and glass beads; thus, differences reported here reflect the base materials and geometries rather than the coating.

### Scanning electron microscopy

The coverslips and aragonite pieces were fixed at room temperature for 10 min in a 4% formaldehyde solution (Sigma-Aldrich), followed by three washes with phosphate-buffered saline × 1 (PBSX1). The samples were then subjected to sequential dehydration using graded ethanol solutions of 50%, 75%, 90%, and 95% for 5 min each, followed by three rinses in 100% absolute ethanol for 10 min each. To complete the dehydration process, the samples were further treated with 100% hexamethyldisilazane (Sigma-Aldrich) for 1 min each, repeated three times. Following dehydration, the samples were mounted onto stubs, coated with gold, and analyzed using a Quanta 200 SEM/ESEM (FEI) scanning electron microscope.

### Image analysis

Image analysis was performed using Fiji. Tissue length was measured along its longest axis with the segmented line tool, while tissue area and solidity were determined using the freehand selection tool and analyzed via shape descriptors. Solidity was defined as the ratio of tissue area to convex area, quantifying deviation from convexity; values near 1.0 indicate compact, well-defined structures, whereas lower values reflect a more spread-out, irregular, or fragmented morphology. To assess tissue–bead interactions, bead density was calculated as the number of beads per mm^2^ of tissue. Bead spatial distribution on glass and aragonite was evaluated using Python libraries (NumPy, Pandas, and Matplotlib). The (x, y) coordinates of each bead were extracted, and pairwise Euclidean distances were computed using the formula: $d=\sqrt{{({x}_{2}-{x}_{1})}^{2}+{({y}_{2}-{y}_{1})}^{2}}$.

### Statistical analysis

Statistical analyses were performed using GraphPad Prism 10.1, with group comparisons conducted using unpaired two-tailed *t*-tests, with a significance level of *α* = 0.05. All quantitative measurements are reported as mean ± SEM.

## Results

**Figure 1.**
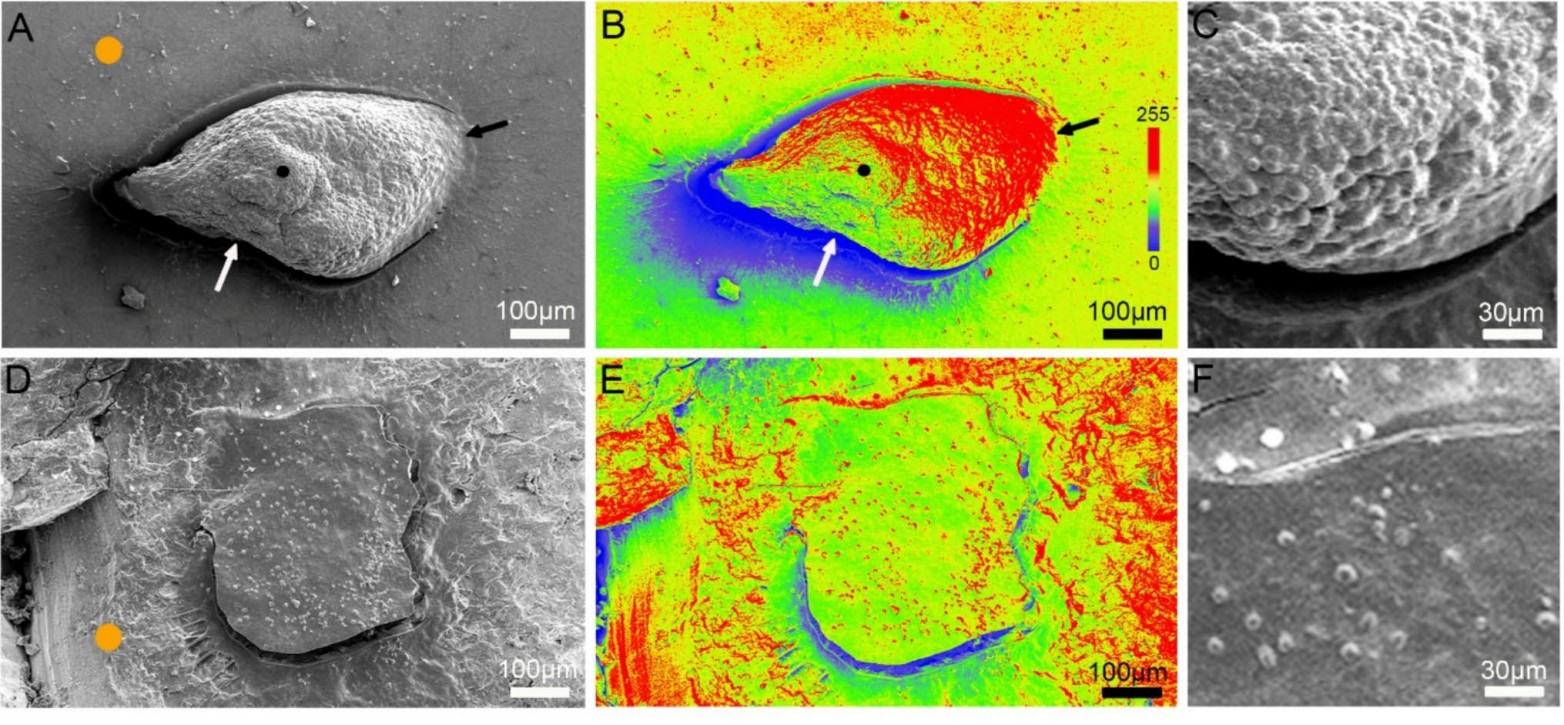
Differential structuring of hippocampal explants on planar substrates. Scanning electron micrographs (SEM) of hippocampal explants cultured for 7 d are shown. (*A*) An explant on a glass substrate (*orange dot*) exhibits an oval shape that extends vertically over two slopes—a steep slope (*white arrow*) and a moderate slope (*black arrow*)—forming a complex three-dimensional structure. The *black dot* marks the top of the explant. (*B*) Color-coded representation of the image in (*A*). (*C*) High-magnification view of the bottom region of the explant. (*D*) An explant on an aragonite substrate (*orange dot*). (*E*) Color-coded representation of (*D*), using the same color scale as in (*B*). (*F*) High-magnification view of the central left region of the explant in (*D*).

The primary objective of this study was to visualize and quantify the structural behavior of injured hippocampal tissue when cultured on flat surfaces with varying adhesion and chemical properties. To achieve this, 0.125-mm^3^ fragments of hippocampi from 1–3-d-old postnatal rat brains were dissected and cultured onto either 12-mm diameter flat glass coverslips or flat surfaces of aragonite crystals. On glass surfaces, the tissue adhered to the substrate adopting a rounded or oval-like shape (Fig. 1*A*). In terms of vertical organization, the tissue on glass displayed irregular height profiles, forming structures with variable slopes and undulating contours, as revealed by image color coding (Fig. 1*B*). The distribution of cell bodies within these volumetric structures was non-uniform, with distinct accumulations forming dense cellular clusters. In certain regions, multi-layered formations exceeded ten cell layers in depth, indicating substantial three-dimensional restructuring within the culture environment (Fig. 1*C*). By contrast, hippocampal tissue fragments placed on a flat aragonite surface exhibited a more pronounced spreading effect compared to those on glass, resulting in a greater degree of flattening (Fig. 1*D*). The tissue spread more extensively across the substrate, resulting in a broader and less compact structure. The three-dimensional organization of the tissue on aragonite was more homogeneous, exhibiting fewer variations in height (Fig. 1*E*). Unlike the irregular and multi-layered 3D architecture observed on glass, tissue cultured on aragonite exhibited a lower volumetric complexity, consisting of only a few layers of cells arranged in a more uniform manner (Fig. 1*F*).

**Figure 2.**
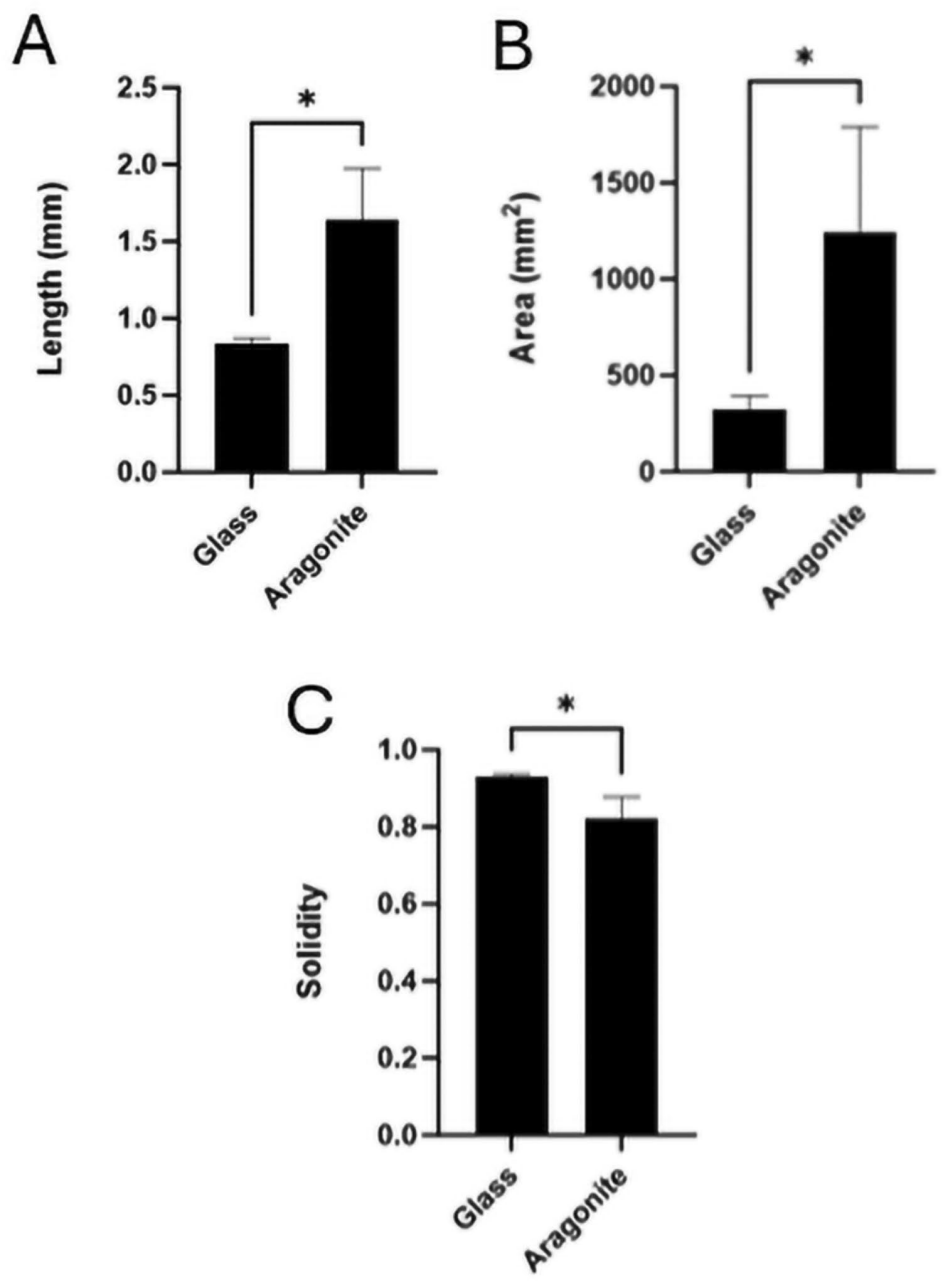
Quantification of tissue morphology on planar surfaces. (*A*) Plot showing the average maximum length of the tissues (*p* = 0.01). (*B*) Plot showing the average area of the substrate-contacting regions of the tissues (*p* = 0.04). (*C*) Plot of the solidity index of the perimeter of these substrate-contacting regions (*p* = 0.02). “Unpaired two-tailed *t*-test”; *n* = 3.

To systematically compare the effects of glass and aragonite surfaces on hippocampal tissue morphology, we measured key structural parameters under both conditions. Analysis of tissue length revealed that while the average length of tissue cultured on glass was 0.83 ± 0.03 mm, the tissue on aragonite was approximately twice as long (1.64 ± 0.33 mm, *p* = 0.01, unpaired two-tailed *t*-test) (Fig. 2*A*). Additionally, the total area occupied by the tissue was significantly greater on aragonite (Fig. 2*B*): the average area covered by tissue on aragonite reached 1.24 ± 0.55 mm^2^, whereas on glass the area was 3.8-fold smaller (0.32 ± 0.73 mm^2^, *p *= 0.04, unpaired two-tailed *t*-test). Furthermore, tissue cultured on glass exhibited a compact, well-defined structure with a smooth, continuous border, remaining confined to a smaller area. In contrast, tissue grown on aragonite appeared more spread out, covering a larger surface while displaying an irregular shape with jagged edges and fragmented regions. These morphological differences were quantitatively reflected in the solidity analysis (Fig. 2*C*), where tissue grown on glass showed significantly higher solidity (0.932 ± 0.005) compared to that on aragonite (0.821 ± 0.055, *p* = 0.02, unpaired two-tailed *t*-test). The lower solidity observed in aragonite-grown tissue indicates its more spread-out nature, irregular borders, and disrupted regions, resulting in a less compact overall structure. Together, these results demonstrate that while glass supports a multi-layered, well-organized, nearly circular 2D base accompanied by irregular 3D structures, aragonite promotes a flatter, more homogeneous 3D morphology that is irregular in 2D due to increased lateral expansion.

**Figure 3.**
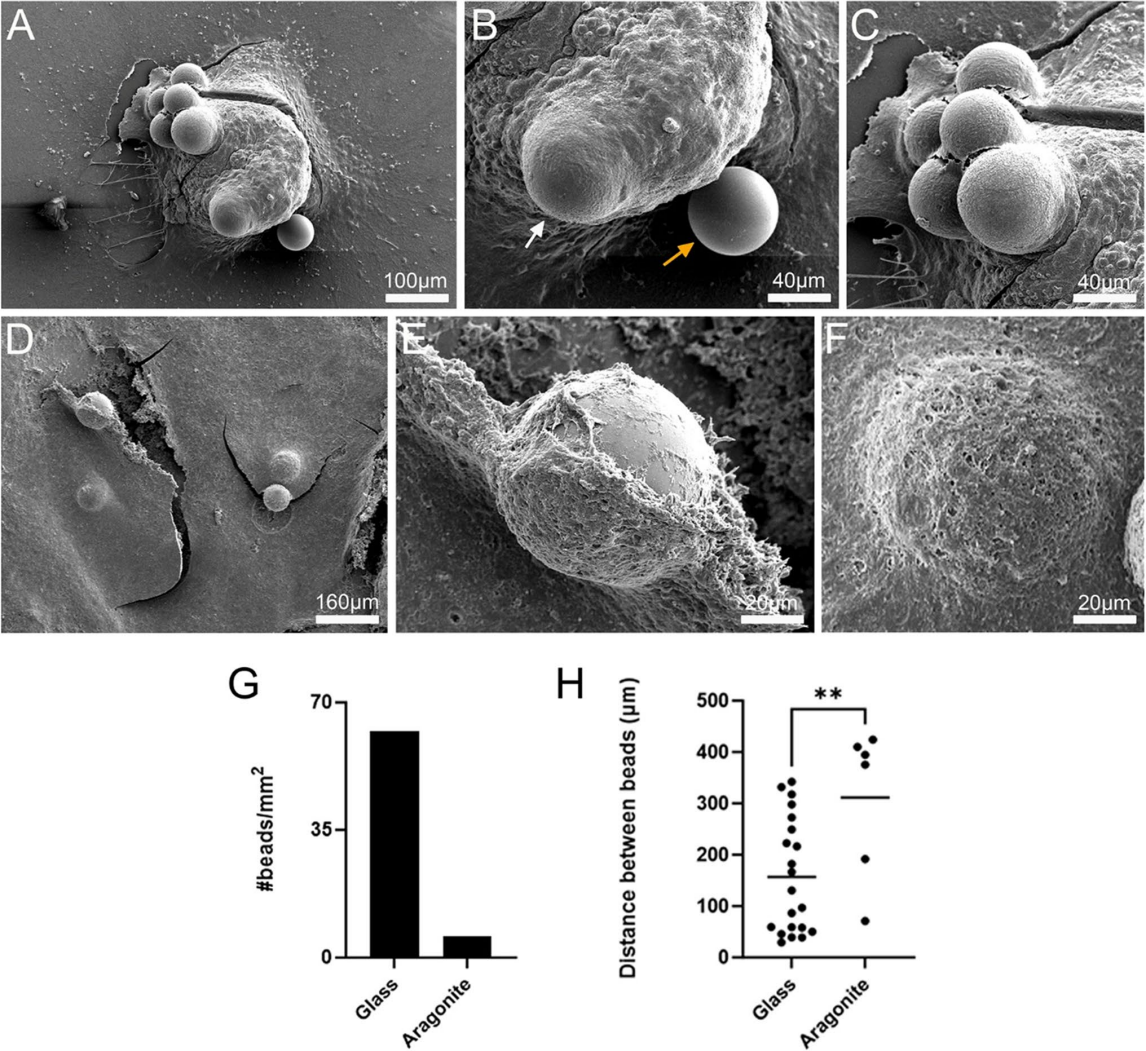
Tissue structural remodeling in response to glass beads. (*A*) SEM image of tissue on a glass substrate with glass beads. Note that the bottom right bead is the only one not encapsulated by the tissue. (*B*) Magnified view of the lower region of the tissue in (*A*), illustrating vertical growth that encapsulates a bead (*white arrow*). The *orange arrow* indicates a controlled, uncovered glass bead. (*C*) Magnified view of the upper left region of the tissue in (*A*), showing a cluster of encapsulated beads. (*D*) Tissue grown on an aragonite substrate, with 4 glass beads encapsulated by the planar tissue. (*E*) Magnified view of the upper left bead in panel (*D*) reveals the tissue’s internal structures near the bead, demonstrating encapsulation both above and below the bead. (*F*) Magnified view of the upper right bead in (*D*), showing a bump in the planar tissue resulting from the complete covering of the bead. (*G*) Comparison of beads’ densities. (*H*) Comparative analysis of distances between pairs of beads (*p* = 0.01; unpaired two-tailed *t*-test).

The next objective of this study was to determine how hippocampal tissue, which exhibited distinct morphological adaptations when grown on different flat substrates, would respond when introduced to a three-dimensional (3D) substrate. To address this, hippocampal tissue fragments were exposed to glass beads (70 µm in diameter), which served as spherical 3D substrates. Direct interaction between the beads and the tissue primarily occurred on the upper surface of the tissue mass (Fig. 3*A*). Notably, in many cases, multiple beads interacted simultaneously contacted a single tissue fragment, creating multiple points of interaction (Fig. 3*A*). The most striking observation was that the presence of glass beads triggered dramatic alterations in the tissue’s 3D morphology. In regions where the tissue interacted with beads, the structure underwent significant vertical deformation, with visible wrinkling, twisting, and elevation of the tissue, often growing upwards toward and around the beads (Fig. 3*B*). In some instances, the deformation was so pronounced that the tissue appeared to partially encapsulate the beads within its structure. Moreover, the spatial arrangement of the beads played a crucial role in determining the final morphology of the tissue. A single bead could induce localized 3D reshaping, while multiple beads in proximity led to the formation of highly intricate and complex 3D structures (Fig. 3*C*).

The response of tissue grown on aragonite to the presence of glass beads differed significantly from that of tissue grown on glass. Unlike the pronounced vertical deformations and complex three-dimensional restructuring observed in the glass-grown tissue, the overall flat morphology of the aragonite-cultured tissue remained largely unchanged in the presence of the glass beads and retained its original planar appearance. However, despite maintaining its flat structure, the tissue actively interacted with the beads, but in a different way than the glass-grown tissue. Rather than growing vertically, the aragonite-grown tissue grew onto the beads and encapsulated them, leading to the formation of localized protrusions or bumps in the otherwise uniform surface (Fig. 3*D*). Bead encapsulation was achieved through gradual tissue bending and overgrowth, where the tissue expanded over and wrapped around the bead (Fig. 3*E*). In some cases, rather than simply bending over the bead, the tissue partially split, with portions of it extending underneath the bead while the rest remained above it (Fig. 3*E*). At the end of this process, the beads were completely encapsulated within the tissue, disrupting the flat geometry and creating discrete dome-like elevations in the structure (Fig. 3*F*). Calculation of bead density revealed that tissue on glass exhibited nearly a tenfold higher density, with 62 beads/mm^2^ compared to 6 beads/mm^2^ on aragonite (Fig. 3*G*). Additionally, the average distance between beads on glass was approximately twofold shorter (157 ± 111 µm) than on aragonite (311 ± 145 µm; *p* = 0.01, unpaired two-tailed *t*-test) (Fig.  3*H*).

**Figure. 4:**
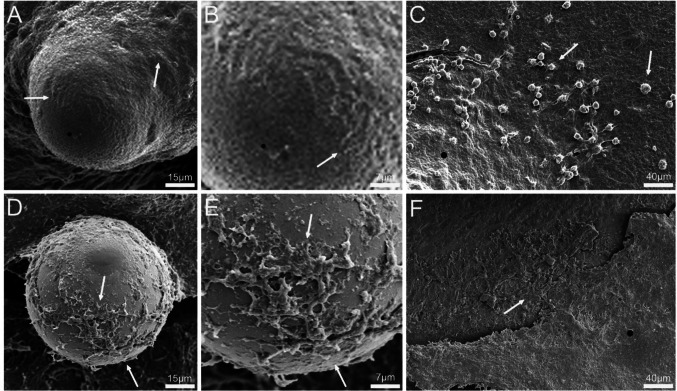
Tissue outgrowth on planar substrates and glass beads. (*A*) Top view of a glass bead covered with tissue. The *right arrow* indicates ascending tissue, while the *left arrow* highlights the tip edges of the climbing tissue. (*B*) Higher magnification of the bead’s top in (*A*), showing the edges of the climbing tissue (*arrow*). (*C*) Cells (*arrows*) and tissue outgrowth emanating from the original tissue (*black dot*) on the planar glass substrate. (*D*) Vertical outgrowth on a glass bead, featuring intact tissue (*bottom arrow*) and tissue extensions (*upper arrow*). (*E*) Higher magnification of (*D*). (*F*) Tissue extension (*arrow*) on the aragonite surface (*black dot*).

The exposure of both types of cultivated neural tissue—those grown on glass and aragonite—to planar and 3D substrates induced an additional shared phenomenon: the outward extension of the tissue onto these matrices through a combination of cellular migration and growth beyond the original tissue mass. This extension was observed on both flat surfaces and glass bead structures, demonstrating the tissue’s ability to actively expand and integrate with its surrounding environment. As shown in Fig. 4*A*, cells from the glass-derived tissue extended outward and almost completely enveloped the glass bead, forming a dense cellular covering. A closer examination revealed multidirectional cellular extensions originating from various points around the bead, converging toward its upper surface (Fig. 4*B*). In addition to growth over the beads, significant tissue expansion directly onto the flat glass substrate was observed (Fig. 4*C*). A similar phenomenon was noted in aragonite-derived tissue, where extending cells migrated onto and covered the surface of the glass beads (Fig. 4*D* and *E*). Additionally, direct tissue expansion onto the aragonite substrate itself was observed (Fig. 4*F*).

These results show that ex vivo hippocampal neural tissue adapts structurally based on the substrate. Glass promoted 3D accumulation, while aragonite flattened the tissue. Glass induced wrapping and deformation around beads, whereas aragonite formed bumps without major shape changes. In both cases, matrix interaction led to tissue extension and growth.

## Discussion

The study aimed to visualize and quantify the structural adaptations of injured hippocampal tissue cultured on two substrates—standard glass and an aragonite crystalline matrix—and to assess responses to 3D cues from glass beads. On glass, tissue fragments adhered to form a circular to oval 2D base with irregular, multi-layered 3D structures. In contrast, on aragonite, the tissue spread uniformly in 3D with enhanced lateral expansion, resulting in an irregular 2D base. Exposure to glass beads induced vertical deformation in tissues grown on glass, whereas aragonite-cultured tissue encapsulated the beads, forming bump-like structures. In both cases, contact with solid structures triggered new tissue formation, highlighting substrate-dependent neural tissue modulation and its implications for neural implant design.

The divergent morphological outcomes of the injured neural tissue observed on glass and aragonite substrates highlight the significant influence of chemical and physical substrate properties on neural tissue organization. Glass coverslips, typically made of borosilicate glass, are characterized by their chemical inertness and the presence of silanol (Si–OH) groups on their surface (Morad 2020). In contrast, aragonite, an orthorhombic polymorph of calcium carbonate (CaCO₃), exhibits a distinct surface chemistry dominated by calcium and carbonate ions, leading to different surface charge and roughness characteristics compared to glass (Tibbitt and Anseth 2009). The ionic composition and crystalline structure of aragonite mediate unique cellular interactions that enhance adhesion and lateral tissue expansion, producing a more uniform, planar morphology (Wang *et al*. 2018; Villanueva-Flores *et al*. 2023).

The solidity of both glass and aragonite provides robust mechanical support by applying uniform pressure on tissues, ensuring homogeneous adhesion and evenly distributed stretching forces across the flat contact area (Weiss 2018; Wasyłeczko *et al*. 2020; Tanikawa 2023; Sutula 2002). Notably, although both substrates maintain a flat interface, the forces transmitted to the surrounding tissue differ. Glass predominantly generates upward forces, resulting in vertical stress that alters the 3D orientation and morphology of portions of the tissue, whereas aragonite exerts a more radial, horizontal influence. This lateral effect may be attributed to the growth-promoting properties of aragonite; combined with its planar solidity, it serves as a matrix that fosters enhanced tissue spreading along wound walls. Furthermore, the stronger adherence of neural tissue to aragonite—as demonstrated in previous findings (Tanikawa 2023), likely limits its ability to undergo significant deformation. In contrast, the weaker adherence to glass allows for greater flexibility and movement, facilitating restructuring toward and around solid structures. These results suggest that the degree of tissue–substrate interaction directly influences tissue adaptability to 3D configurations: higher adherence stabilizes the tissue and prevents deformation, while lower adherence permits greater morphological plasticity in response to external stimuli. Overall, the distinctive mechanical and growth-stimulatory properties of glass and aragonite matrices can be strategically exploited to design implants tailored for specific tissue engineering applications or various wound types.

### Limitations regarding cellular identity of extensions

We observed robust tissue outgrowth on both planar substrates and glass beads. Because the present work focused on substrate-dependent structural remodeling, we did not perform immunophenotyping on the same samples analyzed here. Accordingly, we refrain from assigning a specific cell type to these extensions and refer to them as *cellular extensions/outgrowth*. Definitive attribution will require marker-based assays under identical culture conditions (planned for follow-up work). This clarification does not alter our central conclusion that solid substrates with distinct properties guide tissue-level architecture and outgrowth patterns.

### Response to three-dimensional cues

Our experiments using glass beads to introduce a 3D cue revealed a substrate-specific response. On glass, beads formed multiple focal contacts with the irregular, multi-layered tissue, inducing significant vertical deformation and bead wrapping. This response likely results from mechanotransduction-driven cytoskeletal reorganization and extracellular matrix remodeling (Weiss *et al*. 2020). In contrast, tissue on aragonite remained mostly flat, with cells migrating over and encapsulating beads to form dome-like protrusions. The uniform adhesion and lateral growth promoted by aragonite may limit vertical force transmission, maintaining controlled architecture even when exposed to 3D cues, as seen in hippocampal tissue cultivated on a porous coral skeleton (Sutula 2002). The tissue’s active growth onto and encapsulation of beads demonstrates a robust adaptation mechanism, optimizing surface contact and environmental interaction.

### Functional implications for neural repair

The distinct tissue architectures induced by glass and aragonite have significant implications for neural repair and implant integration. The irregular, multi-layered tissue formed on glass, especially when further shaped by 3D bead interactions, may mimic the complex network topology of the brain, thereby recreating heterogeneous microarchitectures that could enhance tissue connectivity and support restoration following injury (Willerth and Sakiyama-Elbert 2007). However, excessive growth of the tissue into complex 3D formations could pose challenges, such as limiting nutrient diffusion, restricting waste removal, and producing disordered connectivity, something that occurs following TBI or seizure activity in the brain (Yang 2016; Zhuravlev 2000; Zamproni *et al*. 2021).

In contrast, the uniformly flat, laterally expansive tissue formed on aragonite provides a more structured and predictable tissue shape. This architecture promotes controlled cell adhesion and expansion, with minimal disruption to the surrounding neural environment. The lateral organization may also create a more consistent pathway for directional outgrowth, thereby reducing the likelihood of misrouted connections that could impair recovery following injury.

Overall, the contrasting tissue responses to these substrates underscore the need for a strategic approach in neural implant design, where substrate properties are tailored to balance structural complexity, thus optimizing tissue repair.

## Conclusion

In conclusion, this study provides a robust model for examining how the physical and chemical properties of biomaterials govern neural tissue remodeling. In comparing inert glass with a bioactive aragonite matrix, we observed that aragonite promotes a flat, uniformly spreading interface that may support more organized and directional tissue formation, whereas substrates inducing complex 3D architectures, as seen on glass, could be advantageous when enhanced tissue interdigitation is required. These insights underscore the need to tailor implant materials and topographies to specific injury contexts, with significant implications for neural repair. Ultimately, our work paves the way for designing advanced neural implants that promote structural remodeling, thus creating conditions that may facilitate recovery in the injured brain.
